# Endoscopic Ultrasonography in Children with Eosinophilic Esophagitis—A Review

**DOI:** 10.3390/pediatric14010003

**Published:** 2022-01-04

**Authors:** Tomasz Pytrus, Katarzyna Akutko, Anna Kofla-Dłubacz, Andrzej Stawarski

**Affiliations:** 2nd Clinical Department of Paediatrics, Gastroenterology and Nutrition, Medical University of Wroclaw, Skłodowskiej-Curie St. 50/52, 50-369 Wroclaw, Poland; tomasz.pytrus@umw.edu.pl (T.P.); anna.kofla-dlubacz@umw.edu.pl (A.K.-D.); andrzej.stawarski@umw.edu.pl (A.S.)

**Keywords:** eosinophilic esophagitis, children, endoscopic ultrasound

## Abstract

Endoscopic ultrasonography (EUS) is a diagnostic endoscopy of the upper gastrointestinal tract, during which ultrasound of nearby organs is also performed. It is also possible to perform a fine needle aspiration biopsy. Currently, EUS is performed more frequently in adults. Despite some limitations, this diagnostic method is also more and more often performed in pediatric patients. Eosinophilic esophagitis (EoE) is a chronic inflammatory disease of the esophagus, which also occurs in children, and leads to irreversible fibrosis of the esophagus wall, if left untreated. Traditional methods of diagnosing and monitoring EoE treatment have significant limitations, and the use of EUS and total esophageal wall thickness (TWT) assessment may bring measurable benefits. Several studies have shown an increased thickening of TWT in EoE in children compared to pediatric patients with gastroesophageal reflux disease, and a decrease in TWT in adults who responded to EoE treatment. These results suggest that EUS and TWT measurement may become an important test in diagnostics, monitoring the effectiveness of therapy, assessing disease progression, and in individualizing the method and duration of EoE treatment also in children.

## 1. Introduction

Eosinophilic esophagitis (EoE) is a relatively new disease, and the knowledge of this disorder has been developing particularly intensively in recent years. EoE usually occurs in adolescents and young men with a history of food and/or inhaled allergy. The dominant clinical symptoms are swallowing difficulties, incidents of food impaction and symptoms such as those in gastroesophageal reflux disease, which often require differentiation with this disease [1]. In the pathogenesis genetic factors and the occurrence of specific mutations (5q22/TSLP/ and 2p23/CAPN14/) should be considered, which through the modulatory contribution of environmental factors induce the activity of eosinophils, mast cells and proinflammatory cytokines (IL-5, IL-13) in patients with different types of allergies [2]. The diagnosis is based on the characteristic endoscopic mucosal changes, histopathological findings and the eosinophilic infiltration (>15 eosinophils/magnification 400×) found in the esophageal mucosa. This infiltration, on the one hand, causes the thickening and stiffening of the esophageal wall and, at the same time, in a not precisely known way influences the motoric dysfunction observed in manometric examination, especially with the use of high-resolution manometry. These abnormalities result in varied, often stage-dependent symptoms and specific clinical implications. Treatment of EoE involves the so-called 3D-therapy (drugs/diet/dilatation), which consists of an often restrictive, hypoallergenic diet, topically applied or inhaled steroids, and, sometimes, esophageal dilatation in cases of symptomatic strictures. A new experimental method of therapy that refers to the inflammatory mechanism of the disease, related to the production of proinflammatory cytokines (IL-13), is biologic therapy using monoclonal antibodies [3,4]. Currently, monitoring of the effects of treatment consists of a clinical assessment and a resolution of typical clinical symptoms, an endoscopic evaluation of EoE-specific macroscopic changes and a histopathological evaluation. However, the assessment of the effectiveness of therapy or disease progression based only on endoscopic and histopathological examinations may not be sufficient. It seems that the assessment of the thickness and stiffening of the esophageal wall using other imaging techniques may provide significant additional clinical information. Endoscopic ultrasonography (EUS) seems to be very useful, also in children.

## 2. Esophageal Motility in Eosinophilic Esophagitis

The relationship between the occurrence of specific abnormalities of esophageal motility in patients with EoE and their correlation with clinical symptoms is not clear. However, most patients with EoE present various abnormalities of this function. In a meta-analysis by Weiss A.H. et al. [5] in 15 studies of 387 patients, abnormal motility in esophageal manometry was found in 4–87%, and the results presented often remained contradictory. The most common findings in manometric examinations include lack of motor function, abnormal motility, decreased esophageal dilatability/susceptibility, simultaneous contractions, diffuse esophageal spasm, decreased pressure of the lower esophageal sphincter (LES), nutcracker-type changes, or abnormalities such as those seen in esophageal achalasia. Some studies showed a correlation of specific symptoms, e.g., food impaction/dysphagia, with specific motility disorders, whereas others did not show such a correlation. It seems that the essence of motility disorders in children and adults with EoE are biomechanical in nature. In the spatial model the esophagus resembles a cylinder whose structure, wall thickness, compliance, diameter, and internal pressure may induce various motility disorders depending on the stage of the disease. Several esophageal dysfunctions have been demonstrated, especially increased esophageal lumen pressure, decreased compliance and extensibility of the wall, decreased esophageal lumen due to wall edema, and muscular dysfunction (especially in the longitudinal layer). All these abnormalities can be visualized by EUS, especially with the latest generation of equipment [6].

## 3. Endoscopic Ultrasonography in Children

EUS is a procedure during which an endoscopic examination of the gastrointestinal tract and an ultrasound examination of nearby organs are carried out. Additionally, the possibility of performing a fine-needle aspiration biopsy during this procedure makes it more often used in everyday clinical practice, mainly in adults [7]. In addition, the importance of EUS in children is currently growing. This examination has significantly enriched the diagnostic and therapeutic options for many childhood diseases. EUS has proven to be useful in the evaluation and treatment of pediatric conditions including pancreatic and biliary diseases, congenital anomalies, submucosal lesions, cholelithiasis, monitoring inflammatory bowel disease and eosinophilic esophagitis [7,8,9]. Several pediatric studies have demonstrated the high utility of EUS in combination with fine-needle and thick-needle aspiration biopsies, which are technically feasible and relatively safe also in children [7,9]. Therapeutic procedures associated with EUS include endoscopic cystogastrostomy, neurolysis of the visceral plexus, and biliary and pancreatic prosthesis [9]. Indications for EUS in pediatric patients are presented in Table 1.

## 4. Endoscopic Ultrasonography in Children with Eosinophilic Esophagitis

- Usefulness of EUS in the diagnosis of EoE in children

The normal esophageal wall has a layered structure and consists of four layers: mucosa (covered by nonkeratinizing squamous epithelium), submucosa, muscularis propria (circular and longitudinal layer) and appendage. The mucosa consists of two laminas: propria and muscularis. The use of EUS to diagnose and monitor children with EoE is one the new areas of application for this diagnostic technique. This is related to the low availability of this procedure in children and the heterogeneity of the pediatric population (e.g., different age- and height-dependent wall thickness norms). The heterogeneity of the pediatric population related to age and height particularly limits the performance of EUS in younger children, mainly due to lack of appropriate equipment and the need of general anesthesia [7]. However, there are reports in the literature concerning the assessment of the usefulness of EUS in children with EoE. Rabinowitz, S.S. et al. [10] demonstrated that the value of total esophageal wall thickness (TWT) correlates well with the age and height of children, thus the assessment of changes should take this aspect into account. Fox, V.L. et al. [11] used EUS to analyze changes in TWT and respective layers in children with EoE and healthy children showing significant thickening in the EoE group, respectively: TWT (2.8 vs. 2.1 mm; *p* = 0.004), mucosa and submucosa (1.6 vs. 1.1 mm; *p* = 0.001), lamina propria (1.2 vs. 1.0 mm; *p* = 0.043). Interestingly for the circular muscular layer this relationship was not shown.

- EUS in combination with high-resolution manometry

It also seems that EUS in combination with high-resolution manometry may have a significant diagnostic value. Interpretation of these tests together may contribute to a better understanding of the pathomechanisms associated with the occurrence of esophageal motility disorders and the development of clinical symptoms in patients with EoE. The combined use of these two techniques can also significantly improve the diagnostic value of tests and facilitate the decision-making of the diagnosis in patients suspected of EoE. Muroi K. et al. [12] used EUS and high-resolution manometry to analyze patients with EoE and dysphagia in comparison to an EoE group without this symptom. In their analysis, the authors demonstrated a significantly higher value of TWT and dysfunction of the esophageal body function (distal contractile integral—DCI) in the symptomatic EoE patients compared to the asymptomatic group.

- Usefulness of EUS in assessing the effectiveness and choosing the type of EoE treatment in children

In the symptomatic course of EoE in children, macroscopic changes are often found in the endoscopic examination of the upper gastrointestinal tract. The use of steroid therapy in these patients often results in the resolution of clinical symptoms and macroscopic endoscopic finings. This is often used to evaluate the effectiveness of treatment. However, the data in the literature show that achieving clinical remission and involution of endoscopic changes may be insufficient to assess the effectiveness of steroid therapy. It has been shown that achieving clinical and endoscopic remission does not correlate with esophageal TWT reduction. EUS TWT measurements can be useful for a more precise assessment of the effectiveness of therapy. This may contribute to individualization of therapy and adaptation of the length of steroid use for better treatment outcomes [13]. Straumann A. and colleagues analyzed the effect of topical steroids (budesonide) therapy compared to placebo for 50 weeks at a dose of 0.25 mg twice daily on clinical, endoscopic, histological, and esophageal wall parameters measured with EUS in patients with EoE. EUS showed a significant reduction of TWT (3.05 vs. 2.18 mm; *p* < 0.0001), a reduction of mucosal thickness (0.75−0.45 mm; *p* = 0.025), and no change in epithelial thickness (261.22 vs. 277.23 μm; *p* = 0.576) [14]. The effect of steroid therapy mainly affects the mucosal layer, the submucosal and muscular layers are much more resistant to steroids hence, such treatment must be administered for a long time to achieve a sustainable effect [13]. The duration of such treatment is yet undetermined, similarly the duration of the restrictive diet.

Although endoscopic findings in EoE usually involve the area around the lower esophageal sphincter (LES), EUS measurements of esophageal TWT show thickening in both the lower and upper parts [15]. It seems that structural changes in the esophageal body, especially those perpetuated by fibrosis, are responsible for the observed motility disorders despite the disappearance of macroscopic findings in endoscopy, as well as histopathological changes concerning the mucosa.

Another interesting application of EUS is the therapy of benign esophageal strictures e.g., accompanying EoE. The use of EUS in these cases allows to obtain more information about the nature of the stricture, its length and local specificity (which layer of the wall is thickened), being an alternative to classical endoscopy. The study shows that the number of sessions of esophageal dilatation is lower if the mucosa is thickened, higher if the submucosa (muscularis propria) is affected, respectively (1.8 vs. 6.2 sessions; *p* = 0.0002) [16]. The long-term persistence of clinical symptoms in EoE in the form of dysphagia and steroid resistance is mainly related to the infiltration and established fibrosis of the deeper layers of the esophageal wall, primarily the lamina muscularis propria layer. In these cases, endoscopic esophageal dilatation and myotomy have the best lasting results in the treatment [17].

- EUS in combination with free needle biopsy

An unquestionable advantage of EUS is the possibility to perform a targeted fine needle biopsy not only of the mucosa, but also of the deeper layers to evaluate the severity of inflammation and fibrosis [18]. To understand the mechanism of motility disorders and the pathogenesis of clinical symptoms, it seems to be of the utmost importance to determine the phenotype of EoE in each patient. The Dellon E.S. et al. [19] study presents three possible natural phenotypes of the disease: inflammatory, fibrotic, and mixed (inflammatory/fibrotic). The first of these is typical for the pediatric population and the early phase of the disease. Long-term inflammatory changes lead to progressive fibrosis of the esophageal wall, which is irreversible. The risk of fibrosis doubles with each decade and by approximately 5% per year since the diagnosis of EoE was established. Therefore, in adults and children with long-standing EoE, permanent fibrosis-related changes are often present, resulting in decreased esophageal lumen, stiffening of the esophageal wall, and impaired motor function. Monitoring these lesions with EUS and taking targeted biopsies for analysis seems to be extremely important in preventing fibrosis-related complications, for example, by prolonging the duration of steroid therapy and restrictive diet. Such targeted EUS biopsies make it possible to obtain biopsy material from the layers beneath the mucosa, which is essential for the assessment of fibrosis. In children, where the inflammatory type of EoE initially predominates and the lesions are predominantly mucosal, diet and pharmacotherapy are essential in therapy, whereas in adult’s endoscopic dilatation of strictures is often necessary [20].

Carrying out such a method of monitoring the treatment, and due to the possibility of individualized therapy, seems to be very important to prevent the development of irreversible complications. The natural history and long-term follow-up of patients with EoE whose diagnosis was established in childhood indicate two important facts. The Bohm M. et al. [21] study showed that 8 years after diagnosis, only 47% of patients are asymptomatic for EoE, but more importantly 2/3 of them do not receive any specific therapy. Such management increases the risk of fibrosis and stricture formation requiring esophageal dilatation procedures in the future. Early diagnosis and treatment are equally important in preventing complications. Esophageal strictures develop in 17% of patients if diagnosis is delayed <2 years from the first symptoms, in 31% if the delay is 2–5 years, in 38% if the delay is 8–11 years, in 64% if the delay is 14–17 years and in 71% > 20 years from diagnosis [22]. Processes associated with the remodeling of the esophageal wall begin in the early stage of the disease, initially asymptomatic if they are not treated.

Activity of proinflammatory factors in the esophageal wall (esophageal subepithelial activity—ESEA) is crucial for understanding the pathomechanisms involved in fibrosis and the complications in EoE. Inflammation and subsequent fibrosis of the submucosal and muscular layers result in strictures and esophageal motility disorders. In the assessment of this process and monitoring of treatment related to the duration of steroid therapy, it seems crucial to use EUS and taking biopsies from deeper layers in a targeted manner [23]. Japanese researchers have also shown that esophageal motility disorders such as diffuse esophageal spasm, corkscrew esophagus and even esophageal achalasia can be related to EoE, and that an effective treatment can be managed according to EoE treatment standards, often without the need for surgical intervention [24]. These diseases although typical for adults can occur at any age, and EUS is well suited for diagnostic tools in addition to high-resolution manometry. EUS allows the collection of targeted biopsies and the assessment of TWT and specific layers of the esophageal wall.

In the management of EoE, advances in treatment seem to be the new generation of treatment that will have a long-lasting effect on the esophageal mucosa through adhesion and anti-inflammatory effect, as well as other currently used drugs in allergic diseases whose effect is currently not adequately documented. A new commercially available tool for the assessment of esophageal motility disorders especially in terms of lower sphincter function (compliance, diameter) is the Endoluminal Functional Lumen Imaging Probe (FLIP), but due to lack of standards, especially in children, it is currently not widely used [25]. Despite the numerous potential benefits of EUS in children with EoE (Table 2), this method has some significant limitations, as in the case of using EUS for indications other than EoE (the need for sedation, no equipment appropriate for the child’s size) [7]. However, the most important limitation is the lack of validated standards for the esophageal TWT and its layers in children. Therefore, there is a constant need to conduct well-established clinical trials on a large group of patients in order to finally establish the norms and evaluate the usefulness of EUS use in children with EoE.

## 5. Summary

Many questions related to the use of EUS in the diagnosis of EoE in children remain open. Difficulties and ethical concerns are related to the selection of a sufficiently large control group of so-called healthy patients, in whom EUS must be performed under general anesthesia and several biopsy specimens taken for evaluation and comparison with EoE. Hence, the study is usually dedicated only to symptomatic patients with dysphagia or other alarming symptoms. The groups analyzed are generally few, additionally heterogeneous in age, lacking validation and comparative studies. It is also not currently established whether EUS measurements are to be performed only in the lower or middle esophagus or both and how many cm above the LES are to be measured, as well as how to define the middle esophagus. There is also a lack of guidelines, multicenter studies regarding the standards, severity of wall lesions. All these issues need to be clarified.

## Figures and Tables

**Table 1:** Indications for endoscopic ultrasonography in pediatric patients [7,8].

EUS * of the Upper Gastrointestinal Tract	EUS of the Lower Gastrointestinal Tract
Diseases of the biliary tract (i.e., cholelithiasis, choledocholithiasis, biliary strictures) Diseases of pancreas (i.e., acute pancreatitis, recurrent pancreatitis, chronic pancreatitis, autoimmune pancreatitis, drainage of pancreatic cysts) Diseases of esophagus (i.e., eosinophilic esophagitis) Diseases of the stomach (i.e., submucosal lesions, perigastric abscesses) Diseases of the duodenum (i.e., duodenal polyps) Congenital GI anatomic anomalies	Monitoring of inflammatory bowel diseases Bowel lesion Fecal incontinence Encopresis Congenital GI anatomic anomalies

EUS *—endoscopic ultrasonography.

**Table 2:** Potential possibilities of using endoscopic ultrasonography in children with eosinophilic esophagitis.

In diagnostics with the assessment of the thickness of the layers and stiffening of the esophageal wall; In assessing the effectiveness of treatment in children and in individualizing the duration of therapy; Assessment of esophageal motility disorders in the course of EoE * in children (combining with high-resolution manometry—HMR); Assessment of the EoE phenotype (combining with fine needle biopsy); Forecasting the course of EoE in the case of stenosis (combining with fine needle biopsy); Identification of pathomechanisms of EoE development.

EoE *—eosinophilic esophagitis.
